# Autistic Traits Affect P300 Response to Unexpected Events, regardless of Mental State Inferences

**DOI:** 10.1155/2017/8195129

**Published:** 2017-06-04

**Authors:** Mitsuhiko Ishikawa, Shoji Itakura, Hiroki C. Tanabe

**Affiliations:** ^1^Graduate School of Letters, Kyoto University, Kyoto, Japan; ^2^Graduate School of Environmental Studies, Nagoya University, Nagoya, Japan

## Abstract

Limited use of contextual information has been suggested as a way of understanding cognition in people with autism spectrum disorder (ASD). However, it has also been argued that individuals with ASD may have difficulties inferring others' mental states. Here, we examined how individuals with different levels of autistic traits respond to contextual deviations by measuring event-related potentials that reflect context usage. The Autism Spectrum Quotient (AQ) was used to quantify autistic-like traits in 28 university students, and 19 participants were defined as Low or High AQ groups. To additionally examine inferences about mental state, two belief conditions (with or without false belief) were included. Participants read short stories in which the final sentence included either an expected or an unexpected word and rated the word's degree of deviation from expectation. P300 waveform analysis revealed that unexpected words were associated with larger P300 waveforms for the Low AQ group, but smaller P300 responses in the High AQ group. Additionally, AQ social skill subscores were positively correlated with evaluation times in the Unexpected condition, whether a character's belief was false or not. This suggests that autistic traits can affect responses to unexpected events, possibly because of decreased availability of context information.

## 1. Introduction

There is a general assumption that, in order to interact with and have smooth relationships with others in society, one must master certain social behaviors such as empathizing with others, understanding humour, seeing through lies, and comprehending ironies. A diagnosis of autism spectrum disorder (ASD) indicates difficulties with these social behaviors, which are required for understanding others' beliefs, desires, and intentions (termed “theory of mind” [1]).

It has been argued that the foundation of social difficulties may be the absence of a theory of mind [2]. However, Happé [3] revealed that children with ASD can obtain a theory of mind, albeit with some developmental delay. Subsequently, explanations for problems associated with ASD began to focus on the ways that theory of mind is used in social situations. One such explanation involves the idea of context blindness [4], which, for people with ASD, refers to difficulties with the spontaneous use of context in social situations. For instance, to accurately appraise emotions based on another person's facial expressions, we must think about contextual information, such as the events affecting or surrounding the person. Additionally, understanding irony and sarcasm depends heavily on context. There is an example in the Strange Stories test that assesses theory of mind [4]: Emma takes a banana, holds it against her ear, and then says “Look, this banana is a telephone!” In this story, what Emma said is not literally true, but we can understand through context that it is about pretending. Therefore, the use of theory of mind in social situations may be related to the spontaneous use of contextual information.

For the purposes of the context blindness hypothesis, context is defined as not only the flow of linguistic information, but also any type of global information, including that based on visual perceptual. The first global hypothesis states that weak central coherence (WCC [5, 6]) explains deficits in the use of global information in people with ASD. This theory asserts that people with ASD are not inclined to perceive stimuli as a coherent “big picture.” WCC has been examined in some tasks measuring the use of visual and linguistic global information. One of the best known WCC tasks is called the Embedded Figures Test, which requires detection of target shapes within complex big pictures, so it can be expected that individuals with ASD perform better on such a task. Notably, it has been reported that linguistic central coherence was associated with performance in a theory of mind assessment that uses a second-order false-belief task. However, performance on a visual central coherence task was unaffected [7].

The use of linguistic context has also been examined in many studies. Pijnacker et al. [8] conducted an ERP study that examined contextual processing in people with ASD. They found that the N400 component, beginning at 400 ms after stimulus, was diminished in people with ASD compared with their typically developing peers. The N400 is the negative component that reflects semantic language processing, and many studies have reported that the typical N400 can be evoked when semantic deviation in a sentence is recognised [9–11]. Pijnacker et al. [8] argued that people with ASD may show a diminished response to deviation from context. One possible explanation of such a difference is that there is a deficit in the use of context.

The P300 component has also been examined in relation to the cognitive functioning reflected in the N400 [9, 10]. The P300 component had initially been assumed to be related to the recognition of unexpected stimuli [12]. In other words, the P300 can reflect recognition of a violation of expectancy, such that it has been observed in many cognitive tasks such as the oddball task, in which presentation of a so-called “target” or “rare” stimuli randomly mixed with nontarget (to be ignored) distractors. The P300 Speller is one of the most known paradigms used in the Brain Computer Interface [13]. The P300 Speller presents 6 rows and 6 columns containing the set of the characters including an item attended by the observer described as the rare set and the others described as the frequent set [14]. Participants need to focus on one of the items and the sequence of 12 flashes is used to detect the item attended by the observer. In this paradigm, flashing items from the rare set will elicit the P300 component because that is rare event in the context of other flashes. The P300 response is thought to be a positive component that reflects contextual and semantic processing. This component occurs approximately 300–500 ms after stimulus over parietal regions [15].

The oddball paradigm requires only a simple response and comprises relatively simple stimuli, but the P300 response has also been shown during more complex cognitive tasks. For instance, Meinhardt et al. [16] examined the ERP response upon seeing a character acting in either a belief-congruent or belief-incongruent way and found a stronger P300 response in the belief-incongruent condition. The authors suggested from this result that the P300 response could reflect the updating of “a mental model of the environment” and that the P300 amplitude reflects the unexpectedness of the eliciting event. Dunn et al. [17] used ERP to examine how autistic children were influenced by global semantic context. They found that reduced P300 responses were observed in autistic children compared to typically developing children when the target words expected from context were present. Therefore, this diminished P300 response in autistic children may reflect the contextual processing of linguistics.

Recently, the false-belief task was used in a violation-of-expectancy paradigm [18]. The false-belief task requires people to anticipate what a character will do next, which is possible if they have theory of mind and if they understand false belief. Senju et al. [18] reported that people with ASD showed reduced looking times when the character in the false-belief task acted unexpectedly, and they therefore suggested that people with ASD have difficulty using theory of mind spontaneously. However, as mentioned, people with ASD can show deficits in their use of contextual information. Recent theoretical works suggest that autistic behavioral traits can be explained by deficits in forming predictions and processing unexpected events [19–21]. Dungan et al. [22] also suggested the possibility that autistic traits influence how unexpected events are processed, primarily according to context. By measuring the P300, it is possible to know participants' degree of certainty in their predictions from context.

It has been asserted that people without a diagnosis of ASD but showing highly autistic traits may have a similar cognitive style to those with a formal diagnosis and may also experience similar difficulties in social relationships [23]. However, there are many confounding factors in people diagnosed with ASD, including language impairment and sensory hypersensitivity [24], factors that typically have less impact for typically developing people with autistic traits. We focused only on the relationship between individual social difficulties and the processing of contextual information, such that typical university students without a diagnosis were recruited.

Here, we examined whether individuals high in such traits have difficulty using context or thinking about others' mental states. If autistic people are deficient at using context, the P300 component should decrease when a character acts unexpectedly relative to contextual information, and this should occur with or without belief understanding (i.e., use of theory of mind). However, if autistic people show a specific difficulty in apprehending false beliefs and unmet expectations regarding others' mental states, the P300 should decrease only in the unexpected belief condition.

## 2. Materials and Methods

### 2.1. Participants

Twenty-eight university students (15 males and 13 females; age = 19.9 ± 0.8 years) were randomly recruited for this study. All participants spoke Japanese as their first language. The mean Autism Spectrum Quotient (AQ) Japanese version [25] score was 21.89 (range: 10–39, SD = 6.61). This average score is similar to that obtained in other studies that examined the AQ in nonautistic Japanese populations [23]. There was no difference between males (range: 10–39; mean AQ = 21.92; SD = 8.37) and females (range: 11–28; mean AQ = 21.85; SD = 4.537) in AQ total scores. For the AQ, a score of 22 or above is indicative of the broader autism phenotype (subclinical expression of behaviors characteristic of ASD [26]). Prior to analysis, the experimental data were screened for univariate outliers (deviation scores that were more than two standard deviations from the mean) and as a result, one participant was excluded from analysis because this participant did not appear to understand the item content. In studies of autistic traits, it is common to use data only from participants who scored extremely high or extremely low on the AQ (e.g., [27]). To examine group differences, participants were divided into High AQ and Low AQ groups according to the frequency distribution of scores, and around 10 participants from the top and bottom of the AQ score distribution were extracted. The High and Low AQ groups comprised 10 and 9 participants, respectively (score range: 23–33, 4 males, mean AQ_High_ = 27.00, and SD = 3.46; score range: 10–19, 5 males, mean AQ_Low_ = 15.33, and SD = 3.57). As mentioned, there was no gender difference in AQ scores in this study, and we therefore did not further analyse this variable.

All participants were naïve to the purpose of this study, and all reported normal or corrected-to-normal vision. In this study, participants were told that the AQ questionnaire just measures personality traits, and AQ test was done only once. The protocol was approved by the ethical committee of the Psychology Department, Faculty of Letters, Nagoya University, Nagoya, Japan. All participants give their written informed consent to participate in the study.

### 2.2. Apparatus

Visual stimuli were presented through a RDT231WM-X monitor (Mitsubishi, Japan) and were controlled by PsychoPy version 1.83 [28]. EEG data were collected using BrainAmp-DC and ActiCap with 32 channels (Brain Products, Munich, Germany) at standard locations following the extended 10/20 international system referenced to the nose and grounded to the forehead. The sampling rate was 500 Hz, and the impedance was kept below 25 kΩ. The EEG filtering parameter was set to dc-to-70 Hz (12 dB/oct). All EEG data were recorded in a soundproof room using Brain Vision Recorder software from Brain Products.

### 2.3. Stimuli and Conditions

All our stimuli were presented visually. We set up four conditions for stories: No-belief-Expected; No-belief-Unexpected; Belief-Expected; and Belief-Unexpected. All stories comprised five 5-Japanese-word sentences (e.g., *サリーと*/*アンの*/2人が/部屋に/*います*), four sentences of which provided contextual information and the last sentence provided target words. The visual presentation of a “target word,” centered on the monitor, was the ERP onset and determined whether the condition was expected or unexpected. In the Belief conditions (Table 1), two characters were first introduced, and one character placed an object in a given location. A second character was then described as moving the object to a new location. This action was missed by the first character, who initially placed the object. The first character then goes to retrieve the object. In the expected conditions, the first character looks for the object where he or she initially placed it. In the unexpected conditions, the first character looks where the second character placed the object. Additionally, in the No-belief conditions (Table 1), two characters are also introduced, but there is no mental state to infer; the characters are simply described as performing actions in daily situations. Each of the four conditions (Belief-Expected, Belief-Unexpected, No-belief-Expected, and No-belief-Unexpected) was composed of 40 trials, and different sentences were presented for each trial. All sentences were selected from the preliminary test. In the preliminary test, participants were required to evaluate how much the target words were predictable from the story's contextual flow, using a 5-point scale for which only the end anchor points (1 = unpredictable and 5 = predictable) were defined. Unexpected sentences were selected from sentences scored under 3 points; on the other hand, Expected sentences were selected from sentences scored over 3 points.

### 2.4. Assessment

The Japanese version of the AQ [25] was used to measure autistic traits. It comprises 50 items that assess five different areas, with 10 items each: social skills (e.g., finding social situations easy), attention switching (e.g., feeling anxious in new situations), attention to detail (e.g., noticing patterns in things all the time), communication (e.g., enjoying social chit-chat), and imagination (e.g., making up stories easily). Participants were required to choose one of four options (1 = “definitely agree,” 2 = “slightly agree,” 3 = “slightly disagree,” and 4 = “definitely disagree”), and each of the item is scored 1 point if the respondent provides an abnormal response, whether mild or strong. The total score for a participant is hereafter known as the AQ score.

### 2.5. Procedure

After completing the questionnaire, participants performed 160 trials reading different stories, with 40 trials per condition, presented in a random order. Viewing distance was about 60 cm and each Japanese character was about 2° in width and height. All words were presented in a black on white format. The first four sentences of a story were presented initially to provide contextual information. Participants were required to read them once and push the “Enter” key after they understood what was happening in each situation. Next, after the blank (500 ms) and the fixation cross (500 ms) were presented, the last sentence was presented word by word (300 ms each with 200 ms blank), and target word was set to the third word, after 2000 ms from “Enter” key press, across all conditions. Because EEG recordings are affected by participants' fatigue (e.g., body movements), to shorten the experimental time, the remaining words appeared together after the target words were presented for 1500 ms. After the remaining words were presented, participants were required to evaluate how much the last sentence deviated from the story's contextual flow (i.e., they were instructed to rate how much the ending of the story deviated from the rest of the story) as soon as possible, using a 5-point scale for which only the end anchor points (1 = no deviation and 5 = high deviation) were defined. After all tasks were completed, participants were told the purpose of the study. The whole experiment took about 90 minutes.

## 3. Results

### 3.1. EEG Analysis

Raw EEG signals were first filtered between 0.01 Hz (high-pass) and 30-Hz (low-pass) digital filter. The time-locked epochs of 900 ms following the target event were extracted, with a 100-ms baseline. Next, to delete paroxysmal trials caused by large movements or eye blinks, trials showing potentials greater than ±75 *μ*V were removed (5% of each condition). In many past studies, a single electrode has been used to index the P300 response (e.g., [29]). The location is typically *Pz*, which is on the midline and over the parietal region. Therefore, our ROI (region of interest) was only designated as *Pz*. The mean amplitude of P300 on the midline locations (*Pz*, *Cz*, and *Fz*) for each condition was shown in Table 2. Each ERP average contained a minimum of 36 artefact-free trials. Some previous studies have shown differences between ASD and typical development groups only in terms of ERP amplitudes, with no significant difference in ERP latencies [30], so we only analysed ERP amplitudes.

#### 3.1.1. Amplitudes

**Figure 1:**
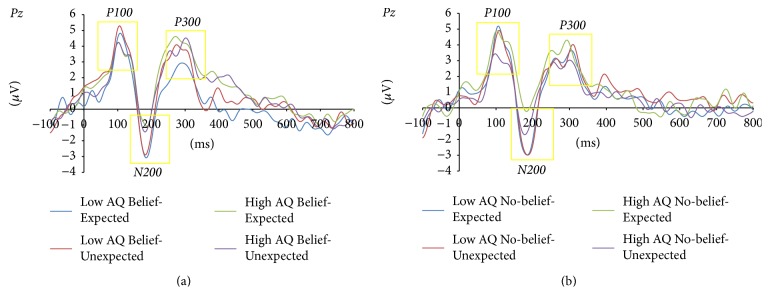
Grand mean ERPs on *Pz* elicited by target words for all conditions. (a) Four conditions in the Belief story condition and (b) four conditions in the No-belief condition.

We took the mean amplitude of the P300 component within the time window of 250–350 ms as the dependent variable. A 2 × 2 × 2 mixed analysis of variance (ANOVA) with two levels of AQ (High and Low), two levels of context condition (Expected and Unexpected), and two levels of belief (Belief and No-belief) was conducted (Table 2). The interaction effect between AQ and context was statistically significant (Figure 1),* F*(1, 17) = 8.731, *p* = .009, *η*_*p*_^2^ = .339. Post hoc* t*-tests corrected by the Bonferroni method indicated that the Low AQ group had larger P300 amplitudes in the Unexpected conditions (SD = 1.712) than in the Expected conditions (SD = 2.521; *p* = .036, *η*_*p*_^2^ = .234). In contrast, the High AQ group displayed marginally significantly smaller P300 responses in the Unexpected conditions (SD = 3.336) (*p* = .076, *η*_*p*_^2^ = .174).

We also examined the mean amplitude of the P100 component within the time window of 50–150 ms as a dependent variable. A mixed 2 × 2 × 2 analysis of variance (ANOVA) with two levels of AQ (High and Low), two levels of context condition (Expected and Unexpected), and two levels of belief (Belief and No-belief) was conducted. The interaction effect for AQ and context was statistically significant,* F*(1, 17) = 4.659, *p* = .045, *η*_*p*_^2^ = .215). Post hoc* t*-tests corrected by the Bonferroni method indicated that the Low AQ group had larger P100 amplitudes in the Expected conditions (SD = 2.094) than in the Unexpected conditions (SD = 1.575; *p* = .052, *η*_*p*_^2^ = .205). In contrast, the High AQ group displayed no significant difference between context conditions (*p* = .366, *η*_*p*_^2^ = .048).

Finally, we took the mean amplitude of the N200 component within the time window of 150–250 ms as a dependent variable. A 2 × 2 × 2 mixed analysis of variance (ANOVA) with two levels of AQ (High and Low), two levels of context condition (Expected and Unexpected), and two levels of belief (Belief and No-belief) was conducted. The interaction effect for context and belief was marginally significant,* F*(1, 17) = 4.292, *p* = .071, *η*_*p*_^2^ = .179. Post hoc* t*-tests corrected by the Bonferroni method indicated that the No-belief condition had larger N200 amplitudes in the Unexpected conditions (SD = 3.039) than in the Expected conditions (SD = 2.629; *p* = .029, *η*_*p*_^2^ = .249). In contrast, the Belief condition displayed no significant difference between the context conditions (*p* = .809, *η*_*p*_^2^ = .004).

#### 3.1.2. Latencies

We took the peak latency of the P300, P100, and N200 component within the time window of 250–350, 50–150, and 150–250 ms, respectively, as the dependent variable. A 2 × 2 × 2 mixed analysis of variance (ANOVA) with two levels of AQ (High and Low), two levels of context condition (Expected and Unexpected), and two levels of belief (Belief and No-belief) was also conducted, respectively. As a result, only the N200 component showed the significant difference. The interaction effect for AQ, context, and belief was marginally significant,* F*(1, 17) = 3.401, *p* = .083, *η*_*p*_^2^ = .167. Post hoc* t*-tests corrected by the Bonferroni method indicated that, in the High AQ group, the Unexpected condition had shorter N200 latencies in the No-belief conditions (SD = 4.878) than in the Expected conditions (SD = 6.910; *p* = .010, *η*_*p*_^2^ = .331). In addition, the interaction effect between AQ and context was statistically significant,* F*(1, 17) = 4.523, *p* = .048, *η*_*p*_^2^ = .210. Post hoc* t*-tests corrected by the Bonferroni method indicated that the Unexpected conditions had shorter N200 latencies in the High AQ group (SD = 5.504) than in the Expected conditions (SD = 4.734; *p* = .003, *η*_*p*_^2^ = .407). Also, the main effect of context was significant,* F*(1, 17) = 6.654, *p* = .019, *η*_*p*_^2^ = .281, and the Unexpected conditions (SD = 3.439) had shorter N200 latencies than in the Expected conditions (SD = 3.999).

### 3.2. Behavioral Results

**Figure 2:**
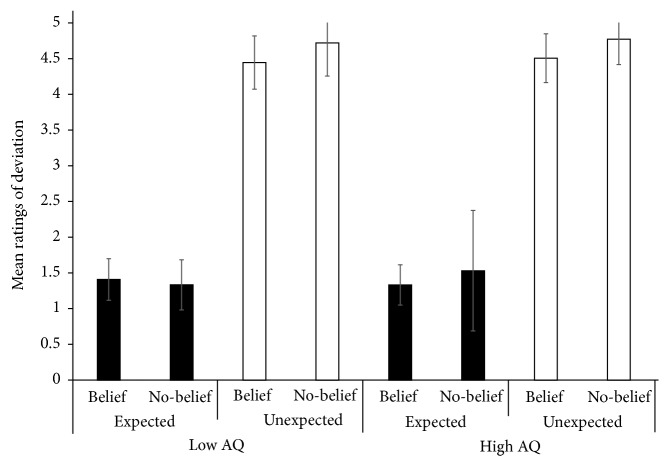
Mean ratings of deviation in all conditions.

A separate ANOVA with participants' ratings of deviation as the dependent variable produced the anticipated significant main effect of context, with the Unexpected conditions evaluated as more deviant than the Expected conditions,* F*(1, 17) = 725.719, *p* < .001, *η*_*p*_^2^ = .977 (see Figure 2). Additionally, a main effect of belief was observed, wherein Belief was evaluated as less deviant than No-belief,* F*(1, 17) = 3.213, *p* = .091, *η*_*p*_^2^ = .159.

**Figure 3:**
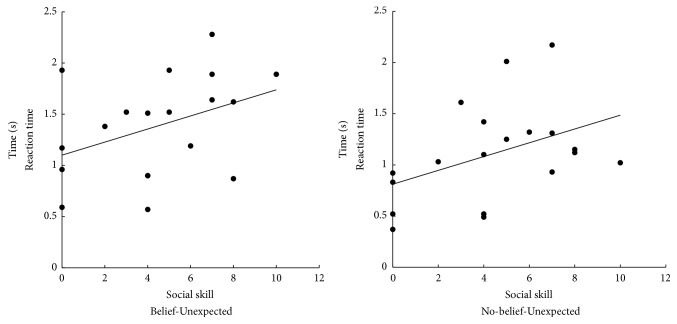
Correlation analysis of social skill and reaction time in the Belief Unexpected condition and in the No-belief Unexpected condition.

The response times for evaluating the level of deviation, measured from the evaluation options presented on the monitor, were also analysed via a 2 × 2 × 2 mixed ANOVA. The context × belief interaction was statistically significant,* F*(1, 17) = 15.151, *p* = .001, *η*_*p*_^2^ = .471. Post hoc* t*-tests revealed that the Belief condition (SD = 0.323) took less time than did No-belief in the Expected condition (SD = 0.474; *p* = .007, *η*_*p*_^2^ = .356). However, the reverse was true in the Unexpected condition: slower performance was seen in the Belief condition (SD = 0.492) than in the No-belief condition (SD = 0.482; *p* = .060, *η*_*p*_^2^ =.193). Additionally, in Belief trials, the Unexpected condition showed slower responses than did the Expected condition (*p* = .002, *η*_*p*_^2^ = .447). To consider the relationship between social difficulties and the use of context at the behavioral level, a correlation analysis was conducted that included both AQ total scores and subscale scores for 19 participants. Both of the Unexpected conditions (Belief and No-belief) were correlated positively only with the social skill subscale of the AQ (*r* = .393, *p* = .096; *r* = .424, *p* = .070; Figure 3). On the other hand, both of the Expected conditions (Belief and No-belief) did not show significant correlation with the social skill subscale of the AQ (*r* = .143, *p* = .560; *r* = .134, *p* = .585). The Expected conditions did not show significant correlations with AQ total score or any of the subscales.

### 3.3. Correlation Analysis between Behavioral and ERP Data

A correlation analysis was conducted between ERP components and behavioral data (ratings of deviation and response times) for 19 participants. Both of the ratings of deviation in Belief conditions (Expected and Unexpected) were correlated with P300 components, respectively (*r* = .555, *p* = .014; *r* = −.549, *p* = .015). No significant correlation was observed in both of the No-belief conditions (Expected and Unexpected) (*r* = −.377, *p* = .111; *r* = .094, *p* = .703). Also, there was no correlation between ERP components and response time.

## 4. Discussion

The purpose of this study was to examine how autistic traits influence responses when contextual deviation is recognised. We also examined whether a nonclinical population that scores high on autistic traits has difficulty using context or inferring mental states.

First, deviation was rated significantly higher in the Unexpected conditions than in the Expected conditions. This result served to confirm that our manipulation of expectations was effective. However, Belief conditions produced higher deviation ratings than did No-belief conditions. In addition, response times for evaluation in the Belief conditions were also slower compared with the No-belief conditions. It may be that the stories that entailed the mental states of others were more complex than stories without the attribution of mental states. The false-belief task has been used to measure linguistic Intelligence Quotient. Happé [3] argued that a certain level of linguistic Intelligence Quotient is required to succeed in the false-belief task, which implies that, all other things being equal, stories that imply another's beliefs should be linguistically more complex and difficult to understand than stories that do not.

In the analysis of evaluation times, regardless of Belief condition, social skill subscores of the AQ were positively correlated in both Unexpected conditions. In other words, people who find it difficult to deal with social situations took longer to evaluate the level of deviation that the stories presented. It may be that this delay is caused by lack of or less than optimal use of contextual information. With sufficient expectancy of contextual flow, the recognition and evaluation of deviation should proceed relatively quickly. Frith [5, 6] argued that, according to WCC theory, people with ASD can be assumed to have lower levels of integration of global contextual information than do non-ASD individuals. This lower level of contextual integration, in turn, produces difficulty that results in slower integration of the sentence flow and slower evaluation times. However, another possible explanation exists. Čeponienė et al. [31] examined the ERP response to deviant sounds in children with ASD. Their results suggested that the children could perceive deviation but that their attention toward it may be inhibited. Relatively low allocation of attention to deviant words could also cause slower evaluations of deviation. However, AQ score was not a significant factor in the ANOVA for evaluation scores. To summarise these results, people with autistic traits can evaluate contextual deviation properly but may require more time to do so. Also, the results of correlation analysis between the participant's ratings of deviant and P300 components showed the significant relationship between the subjective evaluation and ERP components only in Belief conditions. Independently of AQ scores, it has been suggested that P300 may be related with false-belief reasoning [32]. Therefore P300 components may have possibility of predicting the participant's ratings of deviant in Belief conditions.

ERP results indicated that the Low AQ group showed a greater P300 response; on the other hand the High AQ group showed a smaller response when deviation was present. This result revealed that the response to context deviation may be different depending on the level of autistic traits. Gomot and Wicker [33] suggested that the lower P300 response in people with ASD may reflect their weak use of contextual information and lesser inclination to use context for predictions. Bornkessel et al. [34] revealed that the P300 component can be observed when incorrect words in the context of sentences were recognised, and they concluded that P300 may be considered one index of linguistic contextual deviation. Therefore, the P300 response should also be involved in expectancy based on contextual flow in language. This hypothesis has been examined in relation to the linguistic ability of people with ASD. For example, particularly in English speaking countries, the homograph test has been widely used. In this test, where context in sentences has to be actively used to determine proper meanings of words, people with ASD perform more poorly than non-ASD controls (Lopez and Leekman, 2003). Happé [3] revealed that people with ASD have difficulties using contextual information during this test and suggested WCC theory as a framework for understanding such phenomena. WCC theory was first to focus on the reduced use of contextual information among people with ASD. The homograph test requires updating of contextual information as the words of the sentence are presented and prediction of the words that come next. Therefore, worse performance on this test may also suggest that people with ASD have difficulties using linguistic context more generally to build expectancy for what will happen next. This deficit in using context in ASD may also affect the ability to interpret ambiguous sentences, for example, “he bought some glasses” [35]. From our results, supporting context blindness or WCC theory, it can be considered that difficulties using contextual information are related to not only linguistic prediction but also linguistic interpretation. People with ASD has been reported to show language delay, although after their language skills develop they still continue to have communication difficulties in everyday life, such as understanding irony and metaphor [5, 6]. In addition, Happé [3] revealed a relationship between linguistic intelligence and theory of mind. Hence, it can be suggested that using linguistic context may be a cause of the social difficulties in ASD.

We did not have an a priori hypothesis but did analyse P100 and N200 components. Results indicated that the Low AQ group showed a reduced P100 response when deviation was present. In semantic priming tasks, it has been reported that a reduced P100 response is observed when untrained rare words were present [36]. P100 components may possibly reflect semantic processing of words. In the present study, this difference was only observed in the Low AQ group, such that the Low AQ group may predict the target words and realize deviation faster than the High AQ group. However, many studies report that the diminished P100 response in ASD is related to visual attention [37]. Therefore, our results may have been influenced by such a difference. In addition, in the No-belief condition, greater N200 amplitude was observed when deviation was present. We used words showing simple semantical deviation in the No-belief conditions. As a result, it can be considered that deviations in the No-belief conditions may be processed quickly and reflected in the N200 time window. N200, similar to P300, is elicited when participants attend to deviations in oddball paradigms [38]. It has been said that N200 also reflect the recognition of deviation from a mentally stored expectation of the standard stimulus [39]. Although the N400 response reflects semantic comprehension, we could not observe that component in this study. However, P300 response has been claimed to reflect similar cognitive processes to the N400 response [9, 10]. Hence, our P300 response results should be considered to be related to contextual interpretation of semantic processes in people with autistic traits.

Also, the analysis of latencies showed the significant effect of the context only in the N200 component. Shorter N200 latency was observed when deviation was present. Furthermore, the interaction effect for AQ, context and belief was marginally significant and the interaction effect between AQ and context was statistically significant. The post hoc* t*-tests indicated that the significant difference was only observed in the High AQ group No-belief conditions. This result may not be consistent with behavioral result which suggested that people in the High AQ group take longer time to judge context deviation. Frith and Snowling [40] indicated that autistic individuals fail to use semantic context. However, it can be a limitation that some of our sentences did not require using total semantic context. Also, some studies suggested that there may be differences in autistic traits between individuals with and without ASD [41]. Therefore, nonclinical people with autistic traits would have different cognitive styles from individuals with ASD. One possibility is that participants in the High AQ group used some strategies during the task. Recently, broader autism phenotype has been paid more attention [42]. It is necessary to examine the cognitive styles in the nonclinical populations having high autistic traits.

A functional magnetic resonance imaging study conducted by Dungan et al. [22] measured autistic traits and examined how individuals respond to expected or unexpected events. They found that, in the context of an agent's behavior, unexpected versus expected outcomes elicited an especially robust activation of the right temporoparietal junction, and the magnitude of this difference across participants was correlated negatively with autistic traits. Consistent with our study, this study showed that autistic traits can affect the processing of contextual expectations. While our correlational data were based on a small sample, our results are in line with those of previous studies and theoretical works related to autistic recognition.

Our results suggested some cognitive styles in the people having autistic traits; however, the small number of participants can be a limitation. Our results included marginally significant effects; also correlation analysis of AQ scores and behavioral data indicated only marginally significant relations. Although our sample sizes were small to mention firm conclusions, the effect size was seemed to be strong. Further research is required to examine the use of context in people with ASD or nonclinical participants with autistic traits.

Taken together, our findings suggest that the response to deviation is different for those with autistic traits, regardless of whether mental states must be inferred. Although our participants were a nonclinical sample of typical university students, we observed statistically significant differences in ERP responses as a function of relatively low or high levels of autistic traits. Therefore, autistic traits appear to influence recognition of contextual deviation. Furthermore, our results revealed that individuals with autistic traits may not have difficulties in inferring others' mental states, but rather in using contextual information. Level of social skill was correlated with evaluation times, such that the use of context and sociality may be related. Our results supported both WCC theory and the idea of context blindness, and it can be suggested that using contextual information is related to the deficits of sociality and language in ASD.

Recent neuroscientific studies suggest that people with ASD show functional underconnectivity between frontal and posterior brain regions [43]. This lower frontal-posterior synchronization was observed in the sentence comprehension task using linguistic context [44, 45]. Therefore, it is possible that this underconnectively is related to the deficit in using contextual information observed here. However, in this study, we only examined the response to contextual deviation. Therefore, the differential use of contextual information as a function of autistic traits is somewhat speculative. Moreover, our sole focus was linguistic contextual information. Both WCC theory and the idea of context blindness focus not only on linguistic context, but also on more global context. Further research is necessary to clarify the relationship between autistic traits and the use of context.

## Figures and Tables

**Table 1:** Examples of stimulus sentences (target words are italicized).

Condition	Context	Last sentence
Belief-Expected	Sally and Anne are in a room. Sally puts a marble inside the blue box While Sally is away Anne takes the marble from the box and puts inside the red box Then Sally comes back to the room	Sally looks for her marble in the *blue* box

Belief-Unexpected	Sally and Anne are in a room. Sally puts a marble inside the blue box While Sally is away Anne takes the marble from the box and puts inside the red box Then Sally comes back to the room	Sally looks for her marble in the *red* box

No-belief-Expected	Mark wakes up at six. Then he washes his face While Mark washes his face his mother put his breakfast on a table Mark comes to the dining room	After that Mark *pours* a cup of milk

No-belief-Unexpected	Mark wakes up at six. Then he washes his face While Mark washes his face his mother put his breakfast on a table Mark comes to the dining room	After that Mark *burns* a cup of milk

**Table 2:** Mean ERP amplitudes (*μ*V) and latencies (ms) on *Pz*, *Cz*, and *Fz* (standard deviants in parentheses).

	Belief-Expected	Belief-Unexpected	No-belief-Expected	No-belief-Unexpected
P100				
Amplitude (*μ*V)				
Low AQ	2.84 (1.88), 0.27 (1.09), 0.27 (0.75)	3.52 (1.94), 0.26 (0.81), 0.26 (1.11)	3.01 (1.88), −0.02 (0.76), 0.33 (0.74)	2.90 (1.38), 0.61 (0.39), −0.05 (0.57)
High AQ	3.05 (1.68), 0.53 (0.73), 0.02 (0.92)	2.90 (1.43), 0.48 (0.91), −0.11 (0.62)	3.43 (1.50), 0.58 (0.79), −0.16 (0.74)	2.22 (1.18), 0.35 (0.49), −0.32 (0.76)
Latency (ms)				
Low AQ	113 (18.1), 107 (36.0), 92.9 (27.8)	107 (26.4), 113 (28.7), 112 (33.1)	114 (15.5), 107 (27.0), 106 (28.9)	110 (19.5), 110 (27.8), 101 (34.6)
High AQ	101 (32.1), 89.8 (34.1), 99.6 (38.5)	108 (25.1), 111 (34.9), 85 (22.5)	105 (25.3), 107 (27.4), 107 (32.3)	105 (23.5), 93.8 (27.4), 87.4 (38.7)

N200				
Amplitude (*μ*V)				
Low AQ	−0.62 (2.62), −0.2 (1.32), 0.22 (1.01)	0.04 (1.76), −0.15 (1.12), 0.36 (1.42)	−0.35 (1.55), −0.06 (0.87), 0.22 (0.77)	−0.35 (1.55), −0.06 (0.87), 0.35 (0.71)
High AQ	1.11 (2.50), 0.24 (0.96), −0.07 (1.03)	0.76 (1.66), 0.24 (0.80), −0.36 (0.57)	0.33 (2.67), −0.23 (0.87), −0.25 (−.80)	0.33 (2.69), −0.23 (0.87), −0.20 (0.60)
Latency (ms)				
Low AQ	186 (13.3), 186 (26.3), 194 (37.2)	181 (11.4), 181 (21.6), 208 (35.9)	185 (13.8), 179 (17.1), 190 (34.7)	188 (16.4), 296 (28.1), 186 (33.6)
High AQ	189 (23.9) 187 (28.9), 210 (29.5)	187 (25.5), 186 (29.3), 229 (46.0)	204 (27.1), 200 (31.1), 184 (29.7)	181 (14.5), 289 (26.6), 183 (26.7)

P300				
Amplitude (*μ*V)				
Low AQ	4.46 (2.80), 1.52 (0.95). 0.51 (0.68)	5.96 (1.64), 1.78 (0.89), 0.50 (1.36)	5.07 (3.29), 1.10 (0.78), 0.59 (0.68)	5.19 (2.05), 1.40 (0.97), 0.91 (0.63)
High AQ	5.76 (2.81), 2.15 (1.11), 0.03 (1.19)	5.52 (3.25), 2.19 (0.72), −0.13 (0.59)	5.52 (3.87), 1.56 (1.25), 0.36 (0.67)	4.48 (3.69), 1.27 (0.87), 0.65 (0.66)
Latency (ms)				
Low AQ	284 (27.6), 310 (33.8), 284 (44.6)	285 (22.2), 300 (16.8), 263 (34.2)	273 (20.2), 284 (34.1), 283 (36.0)	282 (26.2), 287.6 (40.1), 285 (40.0)
High AQ	288 (27.8), 306 (28.5), 285 (41.1)	287 (39), 301 (22.7), 264 (31.9)	288 (28.7), 303 (20.7), 308 (41.59)	287 (29.6), 298 (32.3), 295 (37.7)
